# Stigma toward mental and physical illness: attitudes of healthcare professionals, healthcare students and the general public in Pakistan

**DOI:** 10.1192/bjo.2020.66

**Published:** 2020-08-03

**Authors:** Muhammad Omair Husain, Syeda S. Zehra, Madeha Umer, Tayyaba Kiran, Mina Husain, Mustafa Soomro, Ross Dunne, Sarwat Sultan, Imran B. Chaudhry, Farooq Naeem, Nasim Chaudhry, Nusrat Husain

**Affiliations:** Division of General Psychiatry, Centre for Addiction and Mental Health; and University of Toronto, Canada; Pakistan Institute of Living and Learning, Pakistan; Pakistan Institute of Living and Learning, Pakistan; Pakistan Institute of Living and Learning, Pakistan; General Adult Psychiatry, South London and Maudsley NHS Foundation Trust, UK; General Psychiatry, Solent NHS Trust, UK; Later Life Psychiatry, Greater Manchester Mental Health NHS Foundation Trust; and University of. Manchester, UK; Department of Applied Psychology, Bahauddin Zakariya University, Pakistan; Department of Psychiatry, Ziauddin Hospital, Pakistan; Department of Psychiatry, University of Toronto; and Centre for Addiction and Mental Health, Canada; Pakistan Institute of Living and Learning, Pakistan; Division of Psychology & Mental Health, University of Manchester, UK.

**Keywords:** Healthcare professionals, mental illness, physical illness, stigma and discrimination, Social Distance Scale

## Abstract

**Background:**

The evidence base for stigma in mental health largely originates from high-income countries.

**Aims:**

This study from Pakistan aimed to address the gap in literature on stigma from low- and middle-income countries.

**Method:**

This cross-sectional study surveyed 1470 adults from Karachi, Pakistan. Participants from three groups (healthcare professionals, healthcare students and the general public) completed the adapted Bogardus Social Distance Scale (SDS) as a measure of stigma.

**Results:**

All three groups reported higher scores of stigma toward mental disorders compared with physical disorders. SDS scores for mental illness in the general public were significantly higher than in healthcare students (mean difference (MD) 6.93, 95% CI 5.45–8.45, *P* < 0.001) and healthcare professionals (MD 6.93, 95% CI 5.48–8.38, *P* < 0.001). However, SDS scores between healthcare students and healthcare professionals were not significantly different (MD 0.003, 95% CI −1.14–1.14, *P* > 0.99). Being female was associated with lower stigma scores and being over the age of 30 years was associated with higher stigma scores.

**Conclusions:**

Stigma campaigns in Pakistan need to target the general population. However, evidence of negative attitudes toward mental illness in healthcare students and healthcare professionals supports the need for stronger emphasis on psychiatric education within undergraduate and postgraduate training in Pakistan.

Individuals suffering from mental and physical disorders not only endure the complications of their illness, but often face negative perception of ill health from society. Stigmatisation encompasses stereotyping, prejudice and discrimination.^1^ Members of a particular group are judged and discriminated against on the very basis of belonging to that group (e.g. gender, race, sexual orientation).^1^ Corrigan has described two types of stigma: public stigma and self-stigma.^2^ Although public stigma refers to society's negative perceptions of an individual deeming them socially unacceptable, self-stigma is the individual's self-labelling as socially unacceptable. Both types of stigma extend to mental illness,^3^ as well as physical conditions such as HIV/AIDS.^4^

Stigma toward mental illness requires attention and understanding, as negative attitudes have persisted despite an increase in tolerance toward other groups.^1^ Stigma has far-reaching consequences, with evidence indicating high levels of stigma correlate with reduced self-efficacy, increased hopelessness, lowered self-esteem and poorer quality of life.^5^ The negative effects of self-stigma on self-esteem and self-appraisal, along with the implications for recovery, are beginning to be explored by the scientific community. The relationship between psychiatric symptoms, stigma and recovery is complex and likely to be bidirectional.^6^ Individuals with greater symptom burden may draw more stigma, and conversely, stigma may worsen existing symptoms of illness.^6^ Therefore, stigma may affect multiple facets of life and general functioning. Stigma in mental health poses a significant barrier to recovery and social inclusion.^7^ Although early intervention in psychiatric illness is associated with improved health outcomes,^8^ stigma has been found to inhibit help-seeking behaviour.^9^ Stigma in mental health is an independent predictor of poorer outcomes after controlling for the initial severity of symptoms and disability.^1^

Negative attitudes held by the general public toward people with mental illness appear to be based on lack of awareness and may largely be shaped by the media.^10^ It is well documented that campaigns increasing awareness and knowledge of mental illness are effective in reducing stigma.^11^ Therefore, one would expect stigma to be less prevalent in healthcare professionals with existing knowledge of mental and physical disorders. Interestingly, evidence shows that negative attitudes toward physical and mental illness in health professionals do not differ significantly from the general public's attitudes.^12^ Negative stereotyping may persist in mental health practitioners despite them having an increased awareness of mental disorders.^13^ A recent world survey found negative attitudes to be more prevalent in low-income countries;^14^ however, the evidence base for stigma in mental health largely originates from high-income settings.

The aim of the present study is to establish whether there are differing attitudes toward mental and physical illness between healthcare professionals, healthcare students and the general public in Pakistan. Attitudes of healthcare professionals can have a significant effect on patient care because negative attitudes contribute to health inequality.^15^ Given their existing knowledge on mental and physical disorders, we hypothesize that negative attitudes toward physical and mental illness in healthcare professionals will be less pronounced than those of healthcare students and the general public.

## Method

This was a cross-sectional study of participant attitudes toward mental and physical disorders. Ethics approval has been obtained from the ethics committee of the Pakistan Institute of Living and Learning (approval no. PILL/ERB/08-09).

### Participants and sampling

The participants were recruited with a convenience sampling technique. Potential participants were approached sequentially, and those meeting inclusion criteria were recruited. The inclusion criteria were age 18 years and over, and belonging to one of the following groups: healthcare students (medical or nursing), healthcare professionals (general practitioners, psychiatrists, nurses or other health professionals) or the general public. Healthcare professionals were recruited from two teaching hospitals, Civil Hospital Karachi and Abbasi Shaheed Hospital, as well as the Primary Care Research and Development Network. Medical and nursing students were recruited from the Dow University of Health Sciences and the Dow Institute of Nursing. The general public sample was recruited from local shopping centres. All participants were briefed about the study by the researchers and informed consent was obtained before surveys were completed. The sample selection was based on convenience method. Given our method of convenience sampling, we acknowledge the limitations of generalisability. The *post hoc* sample size calculation showed that 200 people per group would give results with margin of error (i.e. 95% confidence interval) of ±7%. The *post hoc* power calculation also showed that the sample sizes of all three groups were adequate for detecting a minimum difference of three points on the Social Distance Scale (SDS) with an s.d. of ≤7 points, α of 0.05 and power of 0.8.

### Instrument

The SDS aims to assess the willingness of the respondent to interact with a member of a specific group at different levels of intimacy. The first published SDS was designed by Emory Bogardus to investigate stigma toward different groups based on their occupation, race and religion.^16^ The SDS has been used extensively to measure attitudes relating to a number of psychiatric disorders, including psychotic illnesses^17^ and substance misuse disorders.^18^ The scale has also been used in a number of studies comparing attitudes of healthcare professionals,^19^ and among college students.^20^ Versions of this scale have since been used to examine stigma in mental illness in middle-income countries.^21^ The SDS has also been found to be reliable (α = 0.75–0.9).^22^ The availability of the SDS in the Urdu language informed its use in the current study.

The Bogardus SDS was modified as follows: inclusion of four different mental and physical disorders and use of a three-point Likert scale rather than ‘yes’ and ‘no’ response categories. The questionnaire asked participants about their acceptance of individuals with four mental illnesses (depression, bipolar disorder, substance misuse disorder and psychosis) and four physical illnesses (epilepsy, heart disease, tuberculosis and diabetes) in five different social roles. The five social roles were as a relation by marriage, as a friend, as a work colleague, as a neighbour and as a teacher of their children. Responses were based on a three-point scale to describe the degree of acceptance for each group (will accept, will accept after treatment or will not accept). ‘Will accept’ was assigned a score of 0, ‘will accept after treatment’ was assigned a score of 1 and ‘will not accept’ was assigned a score of 2. A cumulative score for stigma was calculated by the addition of the response scores in all five roles for physical and mental illness, respectively. A maximum total score on the SDS was 40, with higher scores indicating more pronounced negative beliefs.

### Administration of the instrument

All the participants were given information about the purpose of the study. Informed consent was obtained from those willing to take part. Mental disorders were explained by describing symptoms with the Urdu ICD-10.^23^ The SDS and a brief demographic variable sheet were completed by all participants. Confidentiality was assured and all questionnaires were anonymised.

### Statistical analysis

The data was then analysed with SPSS (version 17 for Windows).^24^ The SDS scores for mental and physical illness were compared within each of the three groups, using paired *t*-test to investigate how attitudes differed within group. One-way ANOVA was used to investigate whether there was a difference between the three groups, and *post hoc* tests between pairs of groups (adjusted with Bonferroni correction for multiple testing) were used to identify differences between groups.

Views related to stigma are likely to be affected by age, gender, education and profession. Multiple regression analysis was carried out to investigate the effect of these confounding variables. The SDS scores for mental and physical illness were dependent variables in multiple regression analysis. All independent variables were categorical and used as indicator (dummy) variables. Categories of variables were as follows: profession (three categories: general public, healthcare student and healthcare professional), gender (two categories: male and female), age (three categories: 18–30 years, 31–40 years and >40 years) and education (three categories: no education, primary or secondary education and college or university education).

## Results

The SDS score toward mental illness was 29.82 (95% CI 29.10–30.54) in the general public, 22.89 (95% CI 22.12–23.67) in healthcare students and 22.89 (95% CI 22.28–23.50) in healthcare professionals. The SDS score toward physical illness was 17.95 (95% CI 17.25–18.65) in the general public, 11.75 (95% CI 11.06–12.43) in healthcare students and 12.66 (95% CI 12.15–13.18) in healthcare professionals. We carried out paired *t*-tests for within-group comparisons (Table 1). The difference in the mean score indicates the differences in attitudes toward physical and mental illnesses between the three groups. All three groups reported more negative attitudes toward mental illness (higher mean SDS scores) compared with physical illness. The mean difference between SDS mental and physical illness scores was 11.87 (95% CI 11.08–12.66, *P* < 0.001) for the general public, 11.15 (95% CI 10.51–11.79, *P* < 0.001) for healthcare students and 10.23 (95% CI 9.79–10.67, *P* < 0.001) for healthcare professionals.

**Table 1:** Mean differences within each group for each of the Social Distance Scale (SDS) scores (results of paired *t-*test)

	General public	Healthcare students	Healthcare professionals
SDS mental illness score, mean (95% CI)	29.82 (29.10–30.54)	22.89 (22.12–23.67)	22.89 (22.28–23.50)
SDS physical illness score, mean (95% CI)	17.95 (17.25–18.65)	11.75 (11.06–12.43)	12.66 (12.15–13.18)
Mean difference for SDS mental and physical illness scores within each group (95% CI)	11.87 (11.08–12.66)*	11.15 (10.51–11.79)*	10.23 (9.79–10.67)*

* *P* < 0.001.

**Fig. 1:**
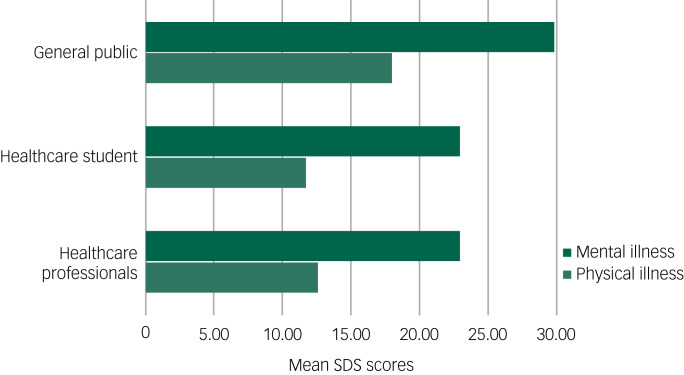
Bar chart of means for Social Distance Scale (SDS) scores for mental illness and physical illness for each group.

We also carried out between-group comparison with one-way ANOVA with Bonferroni adjustment (see Table 2). Mean SDS score for mental illness in the general public was significantly higher than healthcare students (mean difference (MD) 6.93, 95% CI 5.45–8.45, *P* < 0.001), and was also significantly higher than in healthcare professionals (MD 6.93, 95% CI 5.48–8.38, *P* < 0.001). However, mean SDS score for mental illness between healthcare students and healthcare professionals was not significantly different (MD 0.003, 95% CI 1.14–1.14, *P* > 0.99). Mean SDS score for physical illness in the general public was significantly higher than in healthcare students (MD 6.21, 95% CI 4.88–7.53, *P* < 0.001), and was also significantly higher when compared with healthcare professionals (MD 5.29, 95% CI 4.03–6.55, *P* < 0.001). However, mean SDS score for physical illness between healthcare students and healthcare professionals was not significantly different (MD 0.92, 95% CI −1.91 to 0.07, *P* > 0.99). Figure 1 illustrates the mean SDS scores for physical and mental illness in the three groups. The graphs show the highest mean scores on the SDS for physical and mental illness are in the general public group.

**Table 2:** Mean differences across all groups for each of the Social Distance Scale (SDS) scores (results of multiple comparisons from one-way ANOVA after Bonferroni adjustment)

	General public versus healthcare students	General public versus healthcare professionals	Healthcare students versus healthcare professionals
Mean difference for SDS mental illness score across groups (95% CI)^a^	6.93 (5.45–8.45)*	6.93 (5.48–8.38)*	0.003 (−1.14 to 1.14)
Mean difference for SDS physical illness score across groups (95% CI)^b^	6.21 (4.88–7.53)*	5.29 (4.03–6.55)*	0.918 (−1.91 to 0.07)

* *P* < 0.001.

a. *F*(2, 1467) = 73.16, *P* < 0.001.

b. *F*(2, 1467) = 67.24, *P* < 0.001.

We carried out a multiple regression analysis to investigate whether and by how much independent variables such as profession, age, gender and education (after controlling for each other) could predict scores for SDS toward mental and physical illness. The first regression of SDS scores for mental illness on the independent variables showed that the model was significant (*F*(7, 1462) = 34.85, *P* < 0.0001), accounting for 14% of variance (see Table 3). Healthcare students and healthcare professionals significantly predicted a 6.55- and 8.09-point reduction on the SDS, respectively, with the reference category being the general public. Age groups 31–40 years and >40 years significantly predicted a 2.63- and 4.14-point increment, respectively, on the SDS, with the reference category being the 18–39 years age group. Female gender predicted a 0.96-point reduction on the SDS, with the reference category being male. Although the two education groups predicted higher scores, when the reference category was no education, this effect was not significant.

**Table 3:** Multiple regression with Social Distance Scale mental illness score as dependent variable

	Coefficient	s.e.	*t*	*P*>|*t*|	95% CI	
Group						
Healthcare students	−6.549	0.696	−9.410	0.000	−7.914	−5.184
Healthcare professionals	−8.093	0.625	−12.960	0.000	−9.319	6.868
Age category						
31–40 years	2.625	0.555	4.730	0.000	1.536	3.714
>40 years	4.142	0.736	5.620	0.000	2.698	5.587
Gender						
Female	−0.955	0.440	−2.170	0.030	−1.818	−0.092
Education						
Primary or secondary	0.172	1.905	0.090	0.928	−3.565	3.909
College or university	2.763	1.883	1.470	0.142	−0.931	6.458
Intercept	27.477	1.841	14.920	0.000	23.865	31.089

*F*(7, 1462) = 34.85, *P* value for *F* < 0.0001, Adjusted *R*^2^ = 0.1389. Reference categories were as follows: for Group, general public; for age, 18–30 years; for gender, male and for education, no education.

Our second regression model, which investigated the effects of independent variables controlling for each other on SDS scores for physical illness, was also significant (*F*(7, 1462) = 34.85, *P* < 0.0001) and accounted for 11% of the variance (see Table 4). Healthcare students and healthcare professionals significantly predicted a reduction of 6.5 and 5.594 points on the SDS, respectively, when the reference category was the general public group. Age groups 31–40 years and >40 years significantly predicted an increase of 1.37 and 1.98 points on the SDS, respectively, when the reference category was the 18–39 years age group. Females significantly predicted a 1.01-point reduction on the SDS when the reference category was the male group. The two education groups predicted lower SDS scores compared with reference category of ‘no education’, but this effect was not statistically significant.

**Table 4:** Multiple regression with Social Distance Scale physical illness score as dependent variable

	Coefficient	s.e.	*t*	*P*>|*t*|	95% CI	
Group						
Healthcare students	−5.649	0.615	−9.190	0.000	−6.856	−4.443
Healthcare professionals	−5.594	0.552	−10.140	0.000	−6.676	−4.511
Age category						
31–40 years	1.373	0.490	2.800	0.005	0.411	2.335
>40 years	1.975	0.651	3.040	0.002	0.699	3.252
Gender						
Female	−1.099	0.389	−2.830	0.005	−1.861	−0.336
Education						
Primary or secondary	−2.950	1.683	−1.750	0.080	−6.252	0.352
College or university	−1.927	1.664	−1.160	0.247	−5.191	1.336
Intercept	20.072	1.627	12.340	0.000	16.881	23.26369

*F(*7, 1462) = 25.00, *P* value for *F* = 0.0000. Adjusted *R*^2^ = 0.1026. Reference categories were as follows: for group, general public; for age, 18–30 years; for gender, male and for education, no education.

## Discussion

Mental illness is more stigmatised than physical illness in Pakistan across healthcare professionals, healthcare students and the general public. This is consistent with recent findings from high-income settings where mental illnesses are still stigmatised^22^ despite being perceived as ‘diseases as any other’ and are understood to be treatable. Studies from low- and middle-income countries also support greater stigmatisation of mental illnesses.^25^ A survey of medical students and doctors from Pakistan reported that just over half of the participants held negative attitudes toward people with mental illness.^26^ Explanatory models of mental illness with roots in religion and the supernatural are more common in South Asian populations and may contribute to such attitudes.^27^ Mental illness in traditional societies is at times thought to be a consequence of social or moral transgressions, and perceived to be divine punishment, demonic possession or sorcery.^26,27^ When an individual suffering from mental illness lives in a society with these perceptions, they are often subject to shame and social exclusion.^28^ The social implications of stigma affect the patient and extend to the family, whose entire social status comes under threat.^28^ Physical illness is conceptualised as a medical phenomenon, with even somatic symptoms of mental illnesses being considered relatively socially acceptable. However, emotional symptoms are regarded as a sign of weak faith.^29^

Attitudes of the three groups in our sample differed, with stigma toward mental illness being more pronounced in the general public compared with healthcare professionals and healthcare students. We have conducted a rapid review of current evidence and were unable to find comparative published literature exploring the difference in attitudes between healthcare students, healthcare professionals and the general public. However, published literature on stigma has reported that the general public shows a higher degree of social distance to mental illness in comparison with healthcare professionals.^19^ Although there is limited comparative data from Pakistan on the use of the SDS, a study from Nigeria found that moderate social distance toward individuals with mental disorders was commonly reported in university students. However, social distancing was less pronounced in students enrolled in medical studies compared with students of other disciplines.^30^ In Pakistan, medical students have reported more positive attitudes to mental illness compared with their peers from non-medical programmes.^31^ Data from high-income countries suggests that stigma as measured by social distancing is prominent in pharmacy students.^32^ Stigmatising attitudes toward mental illness in healthcare professionals and healthcare students were found to be high in our sample, albeit to a lesser degree than the general public. Studies from low- and middle-income countries have described stigmatising attitudes of health professionals toward mental illness, and it is possible that health education reduces negative perceptions but does not eliminate stigma altogether. Positive attitudes toward mental illness in medical students from Pakistan are thought to be related to the exposure students have to mental health patients as well as their knowledge of mental illness.^31^ A medical explanatory model of mental illness may remove the blame assigned to the affected individual;^33^ however, it may not dispel all the myths surrounding mental illness. It may be pessimism about treatment outcomes that leads to the negative perception of mental disorders. A meta-analysis revealed that although biological explanatory models of mental illness help to reduce the blame attributed to the affected individual, there may be pessimism for recovery.^33^ People with mental illnesses may be perceived as having unchangeable conditions that are fundamentally more serious and persistent.^15^ This perception may lead to greater social distancing and contribute to stigmatising attitudes and stereotyping.^33^ It is therefore important that interventions focusing on raising awareness should include dissemination about the treatability of mental illness.

Regression models showed that being female predicted less stigma toward both mental and physical illness. This is consistent with wider literature, which indicates that women are less likely to desire social distance from individuals with mental or physical illnesses.^34–36^ Similarly, the finding that advancing age is associated with more stigmatising attitudes, both toward physical and mental illness, is supported by published literature.^34,35^ In our study the level of education was not significantly associated with stigma for either type of illness, which is inconsistent with reports that higher levels of education lead to lower levels of stigma.^36^ This may be explained by sparse data in the ‘no education’ category (*n* = 19), but given this finding, interventions to reduce stigma in Pakistan should take this into consideration.

### Strengths and limitations

The strengths of this study are the relatively large sample size and the use of the adapted SDS. The SDS is a scale to assess stigma with good to excellent internal consistency, reliability and construct validity.^22^ However, this study could be further strengthened with other measures that are more commonly used in international research (e.g., measures of attitudes, or more current scales such as the Reported and Intended Behaviour Scale).^37^ Also, caution must be exercised when interpreting the generalisability of our findings, given that recruitment was based on a convenience sampling technique. Furthermore, the sample was restricted to the city of Karachi, the most populous city in Pakistan, which has a relatively higher literacy rate. The attitudes of the wider population in Pakistan, particularly in more rural areas, may differ. Although explanation was provided, this study required the participants to have some existing understanding of various physical and mental health conditions, such as bipolar, psychosis, epilepsy etc.

In conclusion, campaigns to raise awareness about mental disorders are necessary for the general public, healthcare students and healthcare professionals in Pakistan. The presence of negative attitudes toward mental illness in healthcare students and healthcare workers in the current study indicates that focus on psychiatric education needs stronger emphasis in undergraduate and postgraduate training in Pakistan. More formal education about mental disorders and recovery in the curriculum of healthcare students, and clinical exposure to psychiatric care for healthcare professionals, may go some way in tackling stigma in mental health.^38^ Effective evidence-based interventions to address stigma in healthcare professionals in low- and middle-income countries have combined elements of education and direct contact with people experiencing mental illness.^39^ Specifically targeting interventions at healthcare students, who will be the front-line clinical staff of the future, may have a more pronounced effect than mass campaigns geared at the general public. More novel approaches to tackling stigma must also be considered, with media and film representing underused resources. Our group has co-produced the critically acclaimed short-narrative film, ‘DIA’, to bring the unique sociocultural implications of mental health in Pakistan to the fore. ‘Parity of esteem’ must be championed by policy makers at a national level in Pakistan, and learning from government initiatives like ‘Time to Change’ could inform strategies to increase public awareness, challenge negative attitudes and reduce discrimination.^34,40^ The mental health gap is already significant in low- and middle-income countries like Pakistan,^41^ and given that stigma can contribute to disparities in care,^1^ addressing these issues may have a major effect on public health in this setting.

## Data Availability

The data that support the findings of this study are available from the corresponding author, T.K., upon reasonable request.
